# Feline management practices and resource provision in the UK: A questionnaire‐based study of 565 caregivers

**DOI:** 10.1002/vetr.5561

**Published:** 2025-06-07

**Authors:** Samantha Taylor, Isabella M. Mackie, Sarah Heath, Sharmini J. Paramasivam

**Affiliations:** ^1^ School of Veterinary Medicine University of Surrey Guildford UK; ^2^ International Cat Care Tisbury UK; ^3^ Behaviour Referrals Veterinary Practice Chester UK

**Keywords:** cats, diffusers, litter tray, problem behaviour, resources, scratching, toys

## Abstract

**Background:**

Guidelines stating the requirements of an appropriate and healthy feline environment may not always be followed, with resource allocation and distribution misunderstood, reducing welfare and potentially resulting in problem behaviours.

**Methods:**

A 35‐question online survey examining diet, provision of the five pillars of feline environmental needs and problem behaviours was conducted. Analysis was performed to assess recommendation compliance and associations between resource provision and problem behaviours.

**Results:**

Surveys from 565 households were analysed. Most (56.1%) households had two or more cats, and 58.2% of cats had outdoor access. A resting place providing privacy (pillar 1) was provided by 94.5% of households. While key resources were generally provided (pillar 2), deviation from guidance included sharing of food (19.9%) and water (69.4%) bowls in multi‐cat homes and providing fewer litter trays than cats (83.1%). Two‐thirds of households (66.4%) reported problem behaviours, most frequently scratching furniture, which, along with inappropriate urination, was significantly more frequent in multi‐cat homes.

**Limitations:**

The study's design may have resulted in selection bias, and the wording of some survey questions may have been open to misinterpretation.

**Conclusions:**

Adherence to guidance for a feline healthy environment was fragmented in this population, and two‐thirds reported problem behaviours. Further exploration of barriers to adherence and improvements in caregiver and veterinary professional education on appropriate feline environments could reduce problem behaviours.

## INTRODUCTION

According to Cats Protection's ‘CATS Report’ in 2023, 26% of households in the UK own at least one pet cat, with approximately 11 million pet cats in the country.^1^ The way these cats are managed varies across a number of factors, including access outdoors and provision of key resources,^2^ leading to likely different welfare outcomes.^3^ Evidence‐based guidelines from the International Society of Feline Medicine and the American Association of Feline Practitioners detail an appropriate and healthy feline environment using five ‘pillars’: (1) provision of a safe place, (2) appropriate allocation and distribution of key environmental resources (i.e., multiple and separate provision for food, water, toilet, play and resting areas), (3) an opportunity for play and predatory behaviour, (4) consistent and predictable human‒cat social interaction, and (5) an environment that respects the importance of the cat's sense of smell.^4^ In a study of a large group of Australian cats, the pillars were unmet in many households,^5^ and similarly, United States‐based owners were reported to lack knowledge of environmental enrichment of the species.^6^ Such unmet needs may result in stress and problem behaviours,^7^ which can damage the cat‒owner bond and even result in relinquishment, leading to poor welfare outcomes both in the short and long term.^8^, ^9^ Indoor‐only lifestyles^10^ and multi‐cat households^11^ have been reported to be risk factors for problem behaviours, and with both applicable to an increasing proportion of UK cat‐owning homes,^1^ caregiver knowledge of environmental needs becomes even more important. Importantly, creating an appropriate and healthy feline environment does not rely only on the provision of resources, resource type, location and access (which can be affected by social relationships between cats) will also influence use.^3^ Misinterpretation of advice (e.g., placing resources in busy locations in the home deterring use) and deviation from individual cat preferences (forced interactions such as picking up, cuddling, initiating play) could mean that resources are not used and that interactions are negative for the cat. Hence, caregiver education on not only the provision of resources but also on how cats use resources optimally, and how to allow cats to control interactions, is required to improve cat welfare.^4^, ^6^, ^12^

The aim of the present study was to survey cat caregivers in the UK to examine their adherence to the guidance relating to the five pillars of a healthy feline environment, including resource provision (e.g., number and location in the home), caregiver‒cat interactions and scents in the household. Additional aims were to examine the frequency of problem behaviours in the studied group of cats and identify factors associated with the frequency of common problem behaviours.

## MATERIALS AND METHODS

This cross‐sectional study consisted of a survey including 35 questions (Appendix 1) around cat demographics (geographical home locations, sex, breed and origin) and lifestyle factors (number of cats in the home, other pets, outdoor access and diet). Questions specifically related to the five pillars of a healthy feline environment^4^ (provision and location of resting places, water and food bowls, litter trays, other accessories [e.g., toys and cat flaps], caregiver‒cat interactions, diffusers [fragrance, pheromone or other]) were asked, along with questions on observation of problem behaviours and where caregivers go for information on cat care. The questions were inspired and adapted from two previous publications^5^, ^7^ and a literature review, and were modified (grammar and clarity) following a pilot study involving cat caregivers at the University of Surrey. The anonymous survey was designed and distributed using JISC software and was completed by one caregiver per household. The participants met the inclusion criteria if they lived in the UK and if their household included at least one cat. The survey was publicised via social media, with group administrators’ permission, and was open for 3 weeks in 2021. If respondents had multiple cats, where indicated, they were asked to answer based on the cat whose name began with a letter closest to the beginning of the alphabet. Closed‐ended questions were used to determine cat signalment, food/water provision and how the cat was obtained. An ‘other’ option was provided where appropriate to facilitate free‐text responses. The results were analysed and displayed according to the five‐pillar model. To increase response rates, an incentive was offered to participants to enter a draw to win one of eight £25 gift vouchers. The study was part of a veterinary student research project at Surrey University.

### Statistical analysis

The data were downloaded into Microsoft Excel (Microsoft Excel for Microsoft 365 MSO Version 2212 Build 16.0.15928.20278), which was used to perform descriptive statistics for each survey question. Using Jamovi (Jamovi Project Version 2.3.28.0), statistical analyses were conducted on variables relating to the number of cats in the home (multiple/single), distribution of time spent indoors, direct interaction with caregiver and reported problem behaviours. To analyse data that compared groups, for example, multi‐ and single‐cat homes, the chi‐squared test of independence was used. Spearman's correlation was used to analyse relationships, for example, the association between how long caregivers spent petting cats and how much time was spent indoors. Variables reflecting the provision of resources were not included in the analysis (see ‘Limitations’ section for further details).

## RESULTS

A total of 565 surveys were fully completed and hence included in the analysis.

### Respondent demographics

Most respondents were from the south‐east (106/565; 18.8%) or north‐east (98/565; 17.3%) of England, with the fewest responses obtained from Wales (17/565; 3.0%) and Northern Ireland (10/565; 1.8%).

### Signalment and origin of cats

Most cats were neutered (519/565; 91.9%), with an almost even sex distribution (female 288/565; 51%). Most respondents (501/565; 88.7%) knew their cat's breed, and of these cats 425 (75.2%) were non‐pedigree, 74 (14.8%) were pedigree and two were ‘other’. Of the 74 pedigrees, the most common breeds were British Shorthair and Maine Coon (12/74; 16.2% each), followed by Ragdoll (9/74; 12.2%) and Siamese (6/74; 8.1%). Most respondents obtained their cat from a shelter/rescue (221/565; 39.1%) or friend, family or neighbour (109/565; 19.3%), with 77 (13.6%) obtained from online adverts and 61 (10.8%) from a breeder.

### Pets in the household

Multi‐cat homes made up just over half of households (317/565; 56.1%), with the remaining 43.9% (248/565) of households being single‐cat homes. Multi‐cat homes most frequently had two cats (202/317; 63.7%), 54 (17.0%) had three cats, and only 30 (9.5%) and 31 (9.8%) had four or five or more cats, respectively. Most respondents had no pets of other species (341/565; 60.4%), but 75 (13.3%) had one, 40 (7.1%) had two, 37 (6.6%) had three and 72 (12.7%) had four or more other pets.

### Lifestyle: indoor versus outdoor

More than half of the respondents (329/565; 58.2%) reported that their cats spent at least some time outside each day, while 41.8% (236/565) reported that their cats spent all day indoors. For cats with outdoor access, time spent indoors varied between 19 and 20 hours (123/329; 37.4%), 13 and 18 hours (112/329; 34.0%), 7 and 12 hours (74/329; 22.5%), and 0 and 6 hours (20/329; 6.1%). When asked ‘how many hours a day on average are spent in sight of your cat(s)?’, the most frequent answer was more than 8 hours (334/565; 59.1%), followed by 6 hours (136/565; 24.1%), 4 hours (63/565; 11.2%), 2 hours (15/565; 2.7%) or ‘other’ (15/565; 3.0%).

### Adherence to the ‘five pillars’ of a healthy feline environment

#### Pillar one: provide a safe place

Almost all of the respondents reported that their cat(s) had access to a ‘resting location providing privacy’ (534/565; 94.5%).^4^

#### Pillar two: provide multiple and separated resources

##### Diet

Most (489/565; 86.5%) respondents reported feeding a combination of wet (canned, sachets) and dry (kibble) food, most frequently 50% of each (224/565; 39.7%), with only 76 (13.5%) feeding 100% dry food and 33 (5.8%) feeding 100% wet food.

##### Food and water bowls

**FIGURE 1:**
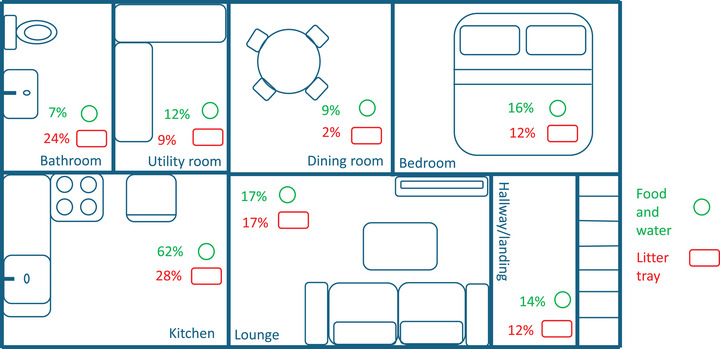
Example house plan illustrating the proportion of respondents reporting locations of food and water bowls and litter trays.

Of the 317 multi‐cat homes, 249 (78.6%) had separate food bowls for each cat, with 63 (19.9%) sharing food bowls and five (1.6%) answering ‘other’ and explaining different scenarios, for example, sharing puzzle feeders for dry food. Over three‐quarters (443/565; 78.4%) of respondents reported providing water in a bowl, while 108 (19.1%) provided a water fountain and eight (1.4%) provided a dripping tap. The remainder provided water in glasses or cups or provided access to rainwater outside. In multi‐cat homes, 220 of 317 (69.4%) cats shared a water bowl. Food and water were provided in a location offering some privacy for 393 of 565 (69.6%) cats. Location within the house was most frequently the kitchen (351/565; 62.1%), followed by the lounge (95/565; 16.8%) and bedroom (88/565; 15.6%) (Figure 1). Food and water were within 50 cm of machinery (e.g., dishwasher, washing machine and boiler) for 172 of 565 cats (30.4%), and 379 of 565 (67.1%) respondents reported that their cat could see their water bowl while eating.

##### Litter trays

Most (462/565; 81.8%) of the respondents provided one or more litter trays, with covered trays and open trays used with similar frequencies (223/462; 48.3% and 215/462; 46.5%, respectively). Further details on the litter provision are provided in Table 1, and the locations are provided in Figure 1. The most frequently reported reason for choosing a litter type was odour elimination (198/462; 42.9%), and the most frequent location was the kitchen (128/463; 27.7%). Most respondents (384/462; 83.1%) answered that the litter tray(s) were not located within 50 cm of machinery (e.g., dishwasher, washing machine and boiler). Only 16.9% (78/462) of respondents provided more litter trays than cats in the home, 43.9% (203/462) provided the same as the number of cats and 27.1% (125/462) provided fewer.

**TABLE 1:** Respondents' answers to questions around litter tray provision, location, litter type and reason for choosing litter.

Question	Respondent answers	Number of respondents (% of the 462 providing litter tray(s))
Type of litter tray(s) provided	Covered (enclosed with a lid or hood)	223 (48.3)
	Open	215 (46.5)
	Other	22 (4.8)
	Automatic self‐cleaning tray	2 (0.4)
Number of litter trays provided	Same as the number of cats in the home	259 (56.1)
	Less than the number of cats in the home	125 (27.1)
	More than the number of cats in the home	78 (16.9)
Type of litter provided	Pellets	157 (34.0)
	Clay	132 (28.6)
	Other	61 (13.2)
	Crystals	40 (8.7)
	I don't know	26 (5.6)
	Recycled paper	24 (5.2)
	Corn	10 (2.2)
	Silica gel	9 (2.0)
Reason for choosing litter	Odour elimination	198 (42.9)
	Cost	83 (18.0)
	Other	52 (11.3)
	Cat preference	31 (6.7)
	Environmentally friendly/biodegradable	27 (5.8)
	Recommended by a friend	25 (5.4)
	Recommended by a veterinarian	18 (3.9)
	Flushable	17 (3.7)
	Easy to clean	13 (2.8)
	Lack of tracking	5 (1.1)
How often is faeces or urine removed from the litter tray?	Multiple times a day	210 (45.5)
	Once a day	160 (34.6)
	Every other day	46 (10.0)
	Other	32 (6.9)
	Once a week	10 (2.2)
How often is the litter tray completely emptied and washed?	Once a week	197 (42.6)
	Every other day	86 (18.6)
	Every other week	74 (16.0)
	Other	59 (12.8)
	Once a day	35 (7.6)
	Multiple times a day	8 (1.7)
Location of litter trays	Kitchen	128 (27.7)
	Bathroom	111 (24.0)
	Lounge	78 (16.9)
	Bedroom	57 (12.3)
	Hallway/top/bottom of stairs	56 (12.1)
	Other	50 (10.8)
	Utility room	41 (8.9)
	Dining room	11 (2.4)
Are litter trays located within 50 cm of machinery (e.g., dishwasher, washing machine, boiler)?	No	381 (82.5)
	Yes	74 (16.0)
	I don't know	5 (1.1)

##### Accessories provided

Most households had a scratching post (476/565; 84.2%), and further information on accessories is included in Table 2. Just over half (182/329; 55.3%) of the households where cats had outdoor access had cat flaps.

**TABLE 2:** Accessories provided within the household.

Accessory	Number of respondents providing accessory (% of 565 respondents)
Cat toys mimicking moving prey (e.g., wand with string)	503 (89.0)
Scratching post	476 (84.2)
Catnip	352 (62.3)
Cat basket	341 (60.4)
Cat tower	312 (55.2)
Cat flap	182 (32.2)
Radiator bed	128 (22.7)
Cat grass	84 (14.9)
Other	35 (6.2)

#### Pillar three: provide opportunity for play and predatory behaviour

**FIGURE 2:**
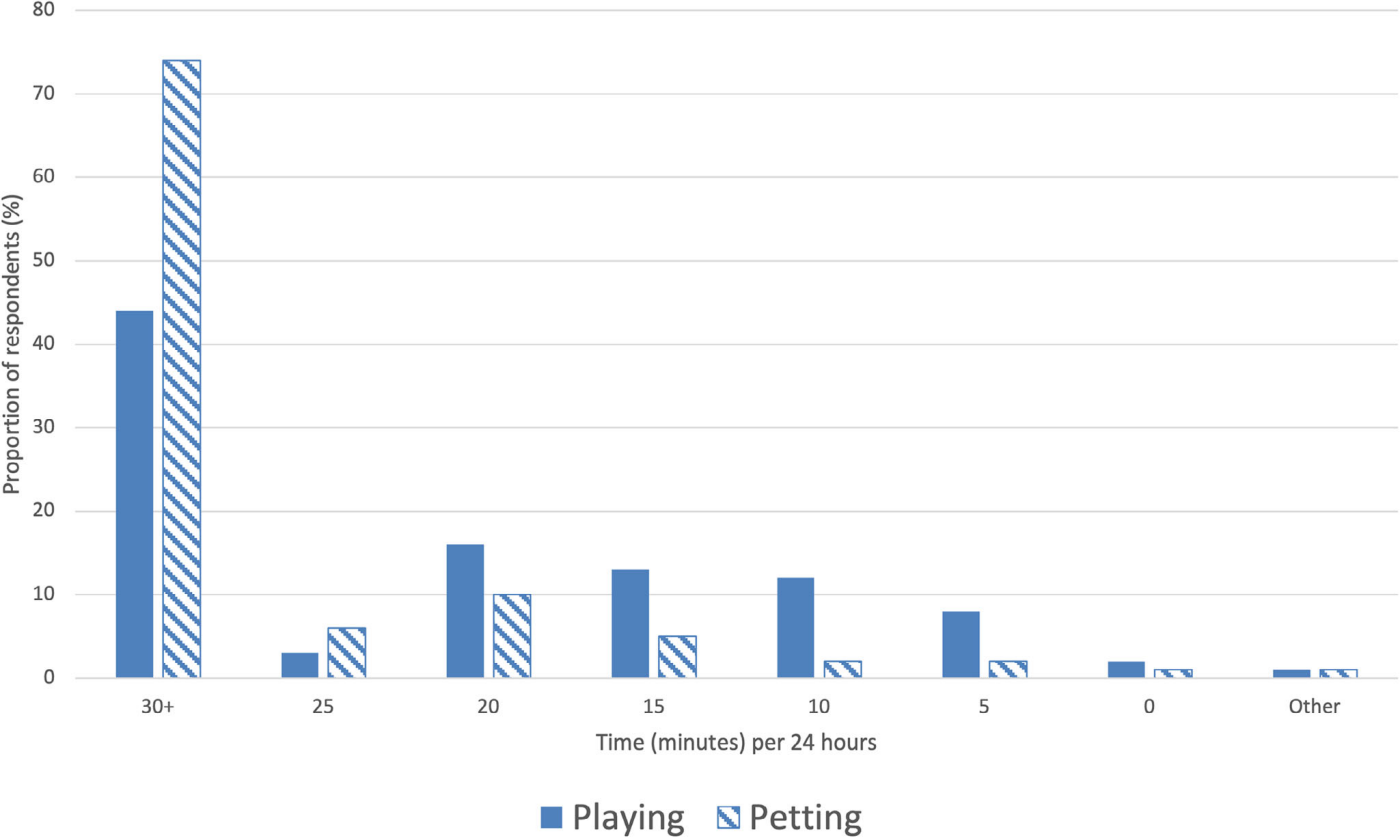
Bar chart of the time that the respondents spent playing with or petting their cat each day.

The majority (503/565; 89.0%) of caregivers provided ‘toys that mimic quickly moving prey (e.g., wand with string)’, but most (279/503; 55.5%) did not rotate toys (at least weekly) to provide novelty. A total of 43.4% (245/565) of caregivers reported playing with their cat for 30 minutes or more per day (Figure 2). Cats that spent more time indoors in a day spent significantly more time in play with their caregivers compared with cats that were given outdoor access (*r*(563) = 0.198, *p* < 0.0010). Additionally, the time spent playing provided by caregivers increased as the number of cats living in the household increased, which was a weak but statistically significant positive correlation (*r*(563) = 0.100, *p* = 0.017).

#### Pillar four: provide positive, consistent and predictable human‒cat social interaction

Among the 565 respondents, nearly three‐quarters (408; 72.2%) reported petting their cat for more than 30 minutes a day, 32 (5.7%) for 25 minutes, 58 (10.3%) for 20 minutes, 27 (4.8%) for 15 minutes and 19 (3.4%) for 10 minutes or less, with 21 (3.7%) answering ‘other’. There was a statistically significant positive correlation, indicating that cats kept indoors for longer periods were petted by their caregivers more (*r*(542) = 0.115, *p* = 0.007).

#### Pillar five: provide an environment that respects the importance of the cat's sense of smell

Over half of the respondents (321/565; 56.8%) reported having a fragrance diffuser (reed, plug‐in, candles or incense), and these were placed in more than one location in 187 (58.3%) of those homes, most frequently in the lounge (231/321; 72.0%), followed by the hallway (123/321; 38.3%), bedroom (121/321; 37.7%), bathroom (96/321; 29.9%) and kitchen (76/321; 23.7%). Only 123 of 565 (21.8%) had a plug‐in diffuser designed to reduce feline ‘stress’ (pheromone or other). Caregivers of multiple cats more frequently had the latter type of diffuser (85/123; 69.1%) than caregivers in single‐cat households (38/123; 30.9%).

### Sources of advice on cat needs

Most respondents (519/565; 91.9%) would seek advice on their cat's physical and emotional needs from a veterinarian (284/565; 50.3%), the internet (170/565; 30.1%), friends/family (81/565; 14.3%), a behaviourist (25/565; 4.4%), ‘other’ and breeder (17/565; 3.0%).

### Problem behaviours

**FIGURE 3:**
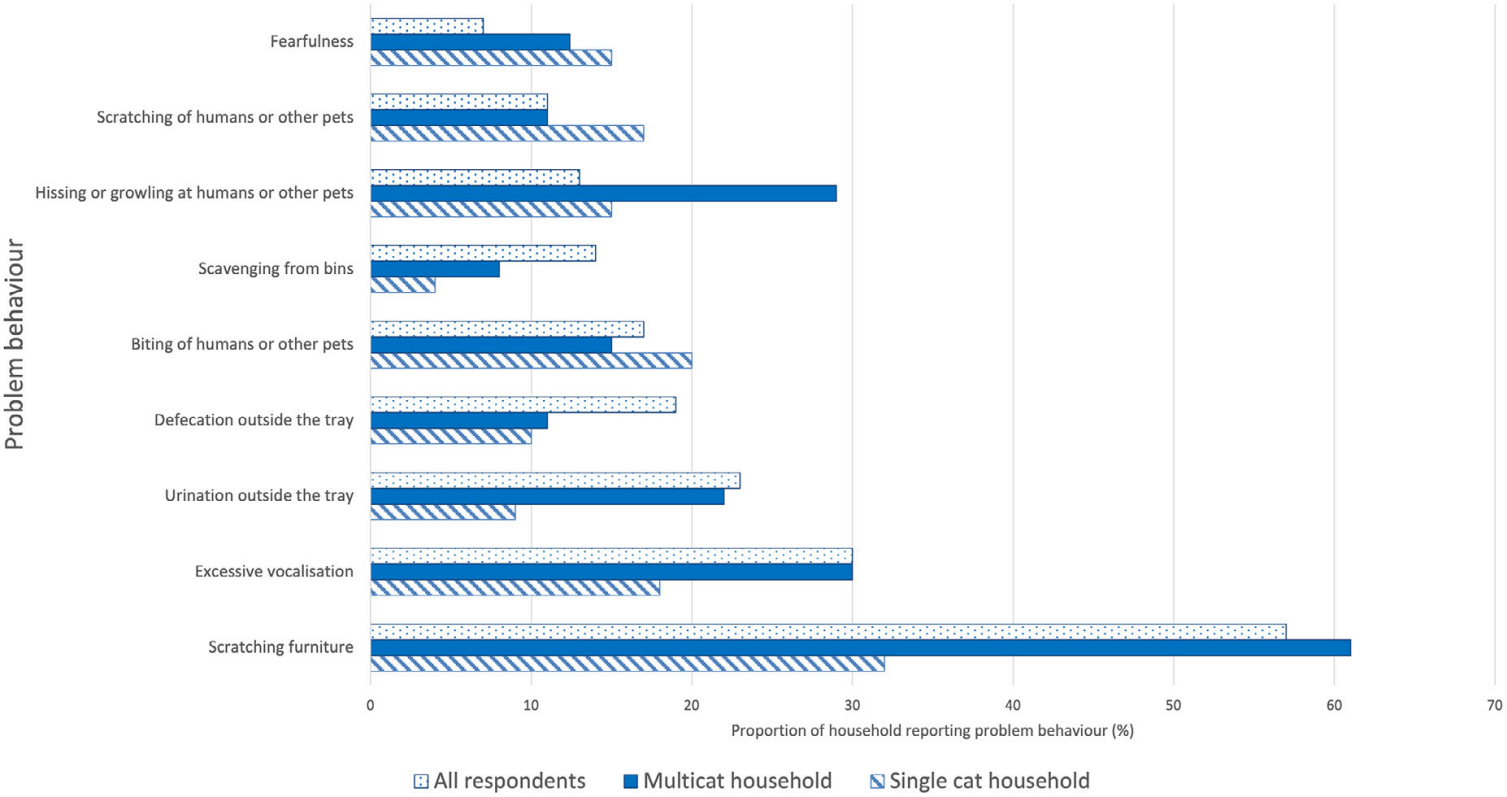
Bar chart of types of problem behaviours exhibited by 371 cats, divided by single‐cat and multi‐cat households.

Two‐thirds of the respondents (375/565; 66.4%) reported that their cat demonstrated at least one problem behaviour. Most respondents reported that their cat demonstrated only one of the listed problem behaviours (173/375; 46.1%), 116 (30.9%) demonstrated two, 49 (13.1%) demonstrated three and 33 (8.8%) demonstrated four or more problem behaviours. The most common problem behaviour was scratching furniture, which was reported by 56.6% (210/371) of respondents, followed by ‘excessive’ vocalisation (110/371; 29.7%), hissing and growling at people or other pets (86/371; 23.2%) and urination outside the litter tray (70/371; 18.9%) (Figure 3).

#### Duration of play and problem behaviours

There was a very slight negative but statistically significant correlation between duration of play and number of behaviour problems reported (*r*(563) = ‒0.091, *p* = 0.030). Cats that were engaged in play with their caregivers for 25 minutes or more a day reported fewer problem behaviours; correspondingly, cats that received 20 minutes or less of play per day reported more problem behaviours.

#### Multi‐cat versus single‐cat homes and problem behaviours

Of the 375 respondents reporting problem behaviours, 158 (42.1%) were living in single‐cat homes and 217 (57.9%) were living in multi‐cat homes. There was no significant difference in the number of problem behaviours between single‐cat and multi‐cat homes (*p* = 0.23). Overall, cats from multi‐cat homes were reported to show problem behaviours proportionately more often than cats from single‐cat homes, but this difference was not statistically significant (*p* = 0.229). Considering each problem behaviour separately, cats from multi‐cat households were significantly more likely to show inappropriate urination (67.1%) than cats from single households (32.9%) (*χ* ^2^(1) = 3.95, *p* = 0.047). Additionally, cats in multi‐cat households were significantly (*χ* ^2^(1) = 6.19, *p* = 0.013) more likely to show furniture scratching behaviour (62.9%) compared to single‐cat households (37.1%). The proportion of cats showing other individual problem behaviours was not significantly different between multi‐cat and single‐cat homes.

#### Indoor versus outdoor lifestyles

Considering each problem behaviour separately, there was no significant difference between indoor and outdoor access groups. However, when cats were kept indoors all day, a higher proportion were reported to display inappropriate urination (37.1%) compared to cats kept indoors for 0‒6 hours (1.4%), 7‒12 hours (12.9%), 13‒18 hours (25.7%) and 19‒20 hours (22.9%) (Figure 3), but these differences were not statistically significant (*p* = 0.581) based on a chi‐squared test of independence.

## DISCUSSION

These survey results provide an insight into resource provision in UK cat‐owning homes and a basis for further study. The respondents met some requirements for pillars of a healthy feline environment; for example, almost all provided a place to rest that offered some privacy, 89.0% provided toys that mimic prey, 84.2% had a scratching post and 72.2% played with their cats for more than a total of 30 minutes a day. However, there were areas of deficiency, including 19.9% of cats in multi‐cat homes sharing food bowls, 69.4% sharing water bowls, 30.4% not provided with privacy to eat and drink, 30.4% with food or water placed near to machinery and 83.1% having equal or fewer litter trays than cats in the home. Two‐thirds of respondents (66.4%) reported problem behaviours, most frequently scratching furniture, despite the provision of scratching posts in 84.2% of the homes. Cats in multi‐cat households were significantly more likely to show inappropriate urination and scratching behaviour than cats in single‐cat homes. Cats receiving 20 minutes or less of play per day displayed more problem behaviours than those played with for longer, and caregivers of cats spending more time indoors spent significantly more time playing with their cats compared with caregivers of cats with outdoor access. These findings suggest that there is scope to improve caregiver education on the provision of the five pillars, potentially improving feline health and welfare. Additionally, the study showed that caregivers frequently seek advice from veterinarians, emphasising the need for them to provide accurate information in an understandable way, and highlighting the need for veterinary students to receive education on species‐specific behavioural needs, which may not currently be adequate in the veterinary curriculum.^13^

The demographics and lifestyles found in the 2023 Cats Protection Report included 33% of households with more than one cat.^1^ The proportion of multi‐cat homes was higher in the present study (56.1%), perhaps due to those completing the survey having increased interest in cats and hence acquiring more than one, but the proportion of those with an indoor lifestyle was similar (37% in the Cats Protection Report and 41.8% in this study). An indoor lifestyle and multi‐cat environment can both present challenges to feline welfare and are risk factors for illnesses such as feline idiopathic cystitis and behavioural problems such as urination outside the litter tray.^5^, ^9^, ^14^, ^15^ In the present study, more respondents with multi‐cat homes reported problem behaviours (57.9% vs. 42.1%), although the difference did not reach significance for all behaviours, only for ‘inappropriate urination’ and ‘scratching behaviour’ (i.e., scratching furniture), which could be behaviours involved in feline communication. However, multiple factors may contribute to problem behaviours,^16^ which may actually be normal behaviours for cats (although not desirable for caregivers); hence, these findings should not be overinterpreted. The small numbers of purebred and entire cats of each breed precluded analysis of these factors that could influence problem behaviours.

Considering the five pillars of a healthy feline environment in turn, as described by Ellis et al.,^4^ we can examine the results of this study in more detail.

### Pillar 1: provide a safe space

This pillar was well met in the current study, as 94.5% of respondents provided such facilities. This finding is similar to that of a study from the United States showing that 92.1% of owners provided a quiet, private hiding space.^6^ However, the present study did not examine this pillar in detail. Caregiver education providing information on more specific aspects of providing a safe place, such as the number of resting places in multi‐cat homes, elevated positions and visual barriers from view,^4^, ^11^, ^17^ could be beneficial.

### Pillar 2: provide multiple and separated key environmental resources

It is important that all owners, particularly those with indoor cats and/or multiple cats, meet environmental needs and provide adequate, separated resources.^18^, ^19^ Most respondents in the current study provided separate food bowls for each cat in multi‐cat homes, but 19.9% reported sharing of bowls, risking the pressure of close contact and removing the opportunity for the cat to eat in privacy, which can be challenging for a species that is solitary in relation to its hunting and feeding behaviours. However, although an adequate number of food (and water) bowls is desirable, this may be simplistic, and cats could use the same food and water bowls but at different times, resulting in low contact with each other.^20^ The sharing of water bowls was even more frequent (69.6%), and water fountains were infrequently provided. As lack of fluid intake can have impacts on urinary tract disease, such as urolithiasis^21^, ^22^ and feline idiopathic cystitis,^23^ every effort should be made to encourage water intake using a variety of techniques to meet each cat's preferences. Food and water bowls were commonly located in busy areas of the house, such as the kitchen, and just under one‐third (30.4%) within 50 cm of machinery. Cats are sensitive to sound^24^ and sudden noise,^25^ so although such locations may be convenient for owners, they may not be optimal for cats. However, the survey did not ask individual questions about food and water location, which may not always be located together.

Inappropriate urination (urination outside the litter tray) was reported by 18.9% of owners of cats with problem behaviours, and defecation outside the tray by 10.8% in the present study, suggesting that indoor elimination is a significant problem behaviour. However, the study could not distinguish marking behaviour from other forms of urination outside the tray, which may have different aetiologies. Litter tray factors such as location, litter aversion, cleaning and sharing of trays can deter use and contribute to elimination outside the tray,^15^, ^26^ and caregiver education on this provision is important. In the present study, most caregivers (80.1%) reported removing faeces or urine at least once a day, with a total cleaning most frequently once a week (45.5%). However, a proportion reported cleaning the tray less frequently, with the presence of urine or faeces in a litter tray potentially deterring litter tray use.^27^ Preference for covered versus open trays may vary between cats (if they are kept clean),^28^ and ideally, a choice of tray type is provided.^29^ Each type was used with similar frequency in the present study. Choice of litter substrate was dictated by odour elimination for 42.9% of respondents, and pellet litter was used most frequently. Odour elimination could drive the use of scented litters or deodorising litter additives (often scented), which may be aversive to cats,^29^ but their use was not asked about in this study. Sand‐like clumping litter is recommended, with pellet‐type litter potentially uncomfortable to stand on, particularly for older cats,^30^ and may make quick removal of urine and faeces more difficult compared to ‘clumping’ litter, as deposits cannot be rapidly removed without changing the whole tray. As with food and water bowls, litter trays were most frequently positioned in kitchens, which can be busy areas, and this also risks closer proximity of litter trays to food and water bowls, which is not optimal for cats.^5^, ^26^ Australian^5^ and American^6^ studies have reported similar findings to the present study in that only a minority of owners (16.9% in this study) provide ‘one litter tray per cat plus one’ as generally recommended (16.9% in this study),^4^, ^26^, ^29^ and 18.2% of respondents did not provide a litter tray at all. While the latter may be acceptable for cats eliminating outside, adverse weather or other cats in the outside environment could mean options to eliminate indoors provide the cat with more choice. Examining barriers to owners supplying more (i.e., the recommended number of) litter trays and managing them appropriately (e.g., reluctance to have more litter trays taking up space, litter without scent) could help improve education and facilitate behaviour change.

Scratching areas are an another important environmental resource (providing visual and scent markers as well as maintaining claw health),^17^ and placing scratching surfaces in more than one location, for example, at places of entry and exit in the home and near sleeping locations can encourage use.^31^, ^32^ In the present study, despite most homes providing scratching posts, scratching furniture was the most frequently reported problem behaviour, and was more common in multi‐cat homes. Examining barriers to providing enough scratching opportunities of variable (preferred) types (e.g., vertical and horizontal, rush matting, sisal rope, carpet, wood) in optimal locations may be beneficial in relation to reducing destructive scratching.^32^

### Pillar 3: provide opportunities for play and predatory behaviours

Offering opportunities to perform predatory behavioural sequences has physical and mental benefits for cats, particularly those kept solely indoors.^4^, ^12^ In the current study, wand‐type toys were frequently provided, but 57.0% of respondents did not rotate toys at least once weekly. Cats may habituate to play objects and fail to be motivated to interact with the toy if it remains the same^33^; hence, caregiver education not only on the provision of toys but also on how to optimally use them is important. Habituation may be less of a concern with wand‐type toys that require caregiver interaction and maintain interest.

### Pillar 4: provide positive, consistent and predictable human‒cat social interaction

Caregiver‒cat interactions are reported frequently in this study, with nearly three‐quarters of respondents reporting that they pet their cat for a total of more than 30 minutes a day. Such interactions can be positive for the cat if broken into shorter interactions, depending on many factors, including genetics and life experiences, but this was not examined in the present study. Caregiver education to avoid forced interactions (e.g., picking up, cuddling, restraining for petting) and allowing the cat to end the interaction may be beneficial.^4^ Time spent playing with cats was significantly longer for indoor cats, emphasising the need for this enrichment for cats without access outdoors, and the time owners spent playing with their cat increased with the number of cats in the home. The latter was a weak correlation, and although respondents were asked to answer for one cat in the home in multi‐cat homes, it is possible that they are including time spent playing with multiple cats, so this result should be interpreted with caution.

### Pillar five: provide an environment that respects the importance of the cat's sense of smell

Given the importance of olfactory communication in this species,^34^ a cat's olfactory environment in the home is likely to be important to their wellbeing. Hence, the additional scents used in 56.8% of homes in the present study may interfere with the cat's olfactory and chemical signalling, which may be challenging.^7^, ^17^, ^35^ Scent diffusers were often located in areas where important resources are provided (litter trays, food and water), preventing cats from avoiding aversive scents when accessing these resources. Less than half the number of respondents using fragrance diffusers in their homes reported having diffusers to reduce feline ‘stress’, but increased use could be of benefit given the evidence that feline pheromone use could reduce behaviours such as scratching furniture, which was commonly reported in this study.^36^

### Limitations

This study had a number of limitations, including the selection of caregivers who can access the internet, who are motivated to complete surveys on cats, who may have more knowledge of feline needs and who may be more reluctant to include answers indicating suboptimal resources. Additionally, the concise nature of the questions prohibited in‐depth exploration of several key areas. For example, differentiating marking/spraying behaviour from urinating outside the litter tray was not possible, and definitions of the problem behaviours were not provided, allowing potential caregiver misinterpretation. Additionally, in the question regarding providing privacy when eating/drinking, it is not clear if this means separate from other cats in the home. Future studies could include annotation of house maps or photographs to allow more accurate analysis of resource provision. The inclusion of answers lacking accurate numbers, for example, more than five cats in the home or more than four litter trays, precluded deeper analysis. It may not have been clear in some questions if respondents should answer for one cat or all cats in the home. Given these limitations, statistical analysis, for example, including resource provision and problem behaviours, and drawing conclusions has been limited to avoid overinterpretation of results and incorrect assumptions. Further surveys of larger numbers of UK cats may build on these findings.

## CONCLUSIONS

The study examined the provision of resources in UK homes with at least one cat, and the results showed that while many homes provided important components of a healthy feline environment, such as private resting places and opportunities to play, resources such as litter trays, water bowls and, less frequently, food bowls often remain shared in multi‐cat homes. These factors can contribute to feline environments being suboptimal from a species‐specific perspective, which in turn may result in physiological stress and predispose individuals to physical illness and problem behaviours. Two‐thirds of owners reported problem behaviours, most commonly scratching furniture. This behaviour, along with inappropriate urination, was significantly more common in multi‐cat homes than in single‐cat homes. Caregivers frequently rely on their veterinarians for information on their cat's physical and emotional health, and it would therefore be beneficial for the veterinary profession to take every opportunity to discuss resource provision with their clients as part of their role in optimising feline health and welfare.

## AUTHOR CONTRIBUTIONS

**Samantha Taylor**: Data analysis; statistical analysis; conception of comparison to pillars concept; referencing (literature review). **Isabella Mackie**: Study design; testing and data collection. **Sharmini J Paramasivam**: Study concept; survey design; statistical analysis; conception of comparison to pillars concept. **Sarah Heath**: Consultation re key behavioural concepts and analysis of problem behaviour data; pillars comparison; referencing (literature review). All authors contributed to the writing of the final paper.

## CONFLICT OF INTEREST STATEMENT

The authors declare no potential conflicts of interest with respect to the research, authorship and/or publication of this article.

## ETHICS STATEMENT

This study followed the University of Surrey's Ethics Committee protocols via the Self‐Assessment for Governance and Ethics and was deemed to not require ethical approval (SAGE‐HDR 640816‐640807‐72071500) on 15 March 2021. The data generated by this survey were fully GDPR compliant, and all personal data were deleted after prize winner selection.

## Supporting information



Supporting Information

## Data Availability

The data that support the findings of this study are available on request from the corresponding author. The data are not publicly available due to privacy or ethical restrictions.
