# Supplementary material for: Feline management practices and resource provision in the UK: A questionnaire‐based study of 565 caregivers
Source: Vet Rec. 2025 Jun 7;197(3):e5561. doi: 10.1002/vetr.5561 (PMC12315624; doi:10.1002/vetr.5561)
Supplement: Supplementary file 1 — Supporting Information [file VETR-197-e5561-s001.docx]

| Question number | Question | Answer style |
| --- | --- | --- |
| 1 | Which region of the UK do you currently live in? | Greater London  East of England  South East  South West  East Midlands  West Midlands  Yorkshire and the Humber  North West  North East  Wales  Northern Ireland  Scotland  Other |
| 2 | What is the gender of your cat? (if you own multiple cats, please answer on behalf of the cat whose name begins with a letter closest to ‘A’ at the start of the alphabet (e.g Alfie and Jake: participants should answer about Alfie) | Female Entire (not neutered)  Male Entire (not neutered)  Female Neutered  Male Neutered  I don’t know |
| 3 | What is the breed of your cat? (if you own multiple cats, please answer on behalf of the cat whose name begins with a letter closest to ‘A’ at the start of the alphabet (e.g Alfie and Jake: participants should answer about Alfie) | Pedigree  Non-Pedigree  I don’t know  Other |
| 3a | If you selected Non-Pedigree, please specify the exact breed | Domestic shorthair (DSH)  Domestic longhair (DLH)  Other |
| 3b | If you selected Pedigree, please specify the exact breed | Persian  British Shorthair  American Shorthair  Siamese  Maine Coon  Ragdoll  Burmese  Sphynx  Devon Rex  Other |
| 4 | How did you obtain your cat? (if you own multiple cats, please answer on behalf of the cat whose name begins with a letter closest to ‘A’ at the start of the alphabet (e.g Alfie and Jake: participants should answer about Alfie) | Shelter / Rescue  Stray / Orphan  Friend  Offspring of a previously owned cat  Gift  Breeder  Pet Store  Online  Other |
| 5 | What is the total number of cats in your household as of today’s date? | 1  2  3  4  5+ |
| 6 | What is the total number of other pets in your household (e.g dogs, birds, fish) as of today’s date? | None  1  2  3  4+ |
| 7 | How many hours per day does your cat spend indoors? | 0-6  7-12  13-18  19-20  All day |
| 8 | What percentage (%) of your cat’s diet consists of wet food? | 0%  25%  50%  75%  100%  Other |
| 9 | Does each cat in your household have its own individual food bowl? | Yes, each cat has its own individual food bowl  No, all cats share food bowls  Other |
| 10 | How do you provide your cat(s) with access to water? | Water bowl  Water fountain  Dripping tap  Other |
| 11 | Does each cat in your household have its own individual water bowl / water source? | Yes, each cat has its own individual water bowl  No, all cats share water bowls  Other |
| 12 | Is your cat able to see its water bowl while it is eating? | Yes  No  I don’t know  Other |
| 13 | Are food and water provided in a location that provides some privacy to your cat while it eats or drinks? | Yes  No  I don’t know  Not applicable |
| 14 | Are food and water provided within 50cm of machinery (e.g dishwasher, washing machine, boiler)? | Yes  No  I don’t know  Not applicable |
| 15 | Where in your household are the food and water bowls located? (please tick all that apply) | Kitchen  Bathroom  Lounge  Bedroom  Dining room  Hallway  Utility room  Other |
| 16 | How many minutes per day on average do you spend playing with your cat(s) | 5  10  15  20  25  30+  Other |
| 17 | How many minutes per day on average do you spend petting your cat(s)? | 5  10  15  20  25  30+  Other |
| 18 | How many hours per day on average do you spend in sight of your cat(s)? | 2  4  6  8+  Other |
| 19 | How many litter trays do you provide in total within your household? | 1  2  3  4+  I do not provide a litter tray |
| 20 | What type of litter tray(s) do you provide within your household? | Open  Covered (enclosed with a lid or hood)  I do not provide a litter tray  I don’t know  Other |
| 21 | What type of litter do you provide within the litter tray(s)? | Crystals  Clay  Recycled paper  Pellets  Silica gel  I dont know  I do not provide a litter tray  Other |
| 22 | Why have you chosen to use this type of litter within the litter tray(s)? | Cost  Odour eliminating  Flushable  Recommended by a friend  Recommended by a vet  I do not provide a litter tray  Other |
| 23 | How often is faeces or urine removed from the litter tray(s)? | Multiple times a day  Once a day  Every other day  Once a week  I do not provide a litter tray  Other |
| 24 | How often is the litter tray completely emptied and washed? | Multiple times a day  Once a day  Every other day  Once a week  Every other week  I do not provide a litter tray  Other |
| 25 | Where in the household are the litter trays located? (please tick all that apply) | Kitchen  Bathroom  Bedroom  Lounge  I do not provide a litter tray  Other |
| 26 | Are litter trays located within 50cm of machinery (e.g dishwasher, washing machine, boiler)? | Yes  No  I don’t know  Not applicable |
| 27 | Does your cat have access to toys that mimic quickly moving prey (e.g a wand with string)? | Yes  No  I don’t know  Not applicable |
| 28 | Are cat toys rotated on a regular basis (at least weekly) to provide novelty? | Yes  No  I don’t know  Not applicable |
| 29 | Which of the following cat accessories (if any) do you provide within your household? (please tick all that apply) | Scratching post (vertical or horizontal)  Cat grass  Cat flap  Cat tower  Cat basket  Radiator hanger bed  Catnip  I don’t know  Other |
| 30 | Do you use plug-in pheromone diffusers in your household (e.g Feliway or Pet Remedy)? | Yes  No  I don’t know  Not applicable |
| 31 | Does your cat have access to its own resting area in a location that provides some privacy? | Yes  No  I don’t know  Not applicable |
| 32 | Do you use fragrance diffusers (reed, plug-in, candles or incense) in your household? | Yes  No  I don’t know  Not applicable |
| 33 | Where in your household do you use these fragrance diffusers? (please tick all that apply, if you do not have any fragrance diffusers in your household, please put ‘not applicable’) | Yes  No  I don’t know  Not applicable |
| 34 | Where would you seek advice on your cats physical and emotional needs? (please tick all that apply) | Breeder  Veterinarian  Behaviourist  Internet  Friends / Family  Pet shop  Not applicable  Other |
| 35 | Has your cat demonstrated any of the following behaviours within the last 12 months? | Scratching furniture  Excessive vocalisation  Inappropriate urination  Defecation outside of the litter tray  Biting of humans or other pets  Scavenging from bins  Hissing or growling at humans or other pets  Scratching of humans or other pets  Fearfulness  No, my cat has not demonstrated any of these behaviours  I don’t know  Other |
